# Alteration of Blood Parameters and Histoarchitecture of Liver and Kidney of Silver Barb after Chronic Exposure to Quinalphos

**DOI:** 10.1155/2015/415984

**Published:** 2015-10-08

**Authors:** Golam Mohammod Mostakim, Md. Mahiuddin Zahangir, Mahbuba Monir Mishu, Md. Khalilur Rahman, M. Sadiqul Islam

**Affiliations:** ^1^Department of Fisheries Biology & Genetics, Bangladesh Agricultural University (BAU), Mymensingh 2202, Bangladesh; ^2^Freshwater Station, Bangladesh Fisheries Research Institute (BFRI), Mymensingh 2201, Bangladesh

## Abstract

Quinalphos (QP) is commonly used for pest control in the agricultural fields surrounding freshwater reservoirs. This study was conducted to evaluate the chronic toxicity of this pesticide on blood parameters and some organs of silver barb,* Barbonymus gonionotus*. Fish were exposed to two sublethal concentrations, 0.47 ppm and 0.94 ppm, of QP for a period of 28 days. All the blood parameters (red blood cell, hematocrit, and hemoglobin) and blood glucose except for white blood cells decreased with increasing concentration of toxicant and become significantly lower (*p* < 0.05) at higher concentration when compared with control. The derived hematological indices of mean corpuscular volume, mean corpuscular hemoglobin, and mean corpuscular hemoglobin concentration were equally altered compared to control. Histoarchitectural changes of liver and kidney were observed after exposure to the QP. Hypertrophy of hepatocytes, mild to severe necrosis, ruptured central vein, and vacuolation were observed in the liver of treated groups. Highly degenerated kidney tubules and hematopoietic tissue, degeneration of renal corpuscle, vacuolization, and necrosis were evident in the kidney of treated groups. In conclusion, chronic exposure to QP at sublethal concentrations induced hematological and histological alterations in silver barb and offers a simple tool to evaluate toxicity derived alterations.

## 1. Introduction

Synthetic pesticides used for controlling pests in agriculture are one of the major causes of aquatic pollution. Sometimes pesticides are directly applied in water bodies for controlling pests and vectors but their residues mostly reach into aquatic ecosystems through surface run off and affect the health of nontarget organisms including fish. Among synthetic pesticides, organophosphates are widely used in agriculture and in health and hygiene programs due to their high effectiveness as insecticide but less persistence in the environment. They are favoured over organochlorines which have long persistence and consequently easily bioaccumulate in food chain. The shift from organochlorines to organophosphates has resulted into increased occurrence of organophosphates into water bodies causing acute and chronic toxicity to fish fauna [1].

Quinalphos 25EC (QP) is an organophosphate extensively used as a pesticide [2]. It is classified as a yellow label (highly toxic) pesticide in the Indian subcontinent and extensively used in agriculture for protection of variety of crops, such as wheat, rice, coffee, sugarcane, and cotton. It is a hard pesticide, which has become a matter of concern because of its potential and hazardous effect. Effects of quinalphos on histopathological alterations were studied in the vital organs like brain, gill, and liver [3], in respiratory rate and food consumption [4], in neurobehavioral responses [5] of* Cyprinus carpio*, and so forth.

Normally, most aquatic animals including fishes respire through their gills and sometimes with the help of skin. These respiratory organs frequently encounter hazardous pollutants which are present in water in different forms and these pollutants may lead to the alteration in the normal area which causes the reduction in oxygen consumption and physiological imbalance in the organism. Today the role of histopathology has received a significant interest as an endpoint in endocrine disrupting chemicals research in aquatic organisms, because histopathological changes are often the result of the integration of a large number of interactive physiological processes. The frequent occurrence of organophosphate pesticides has been regarded as a serious global public health problem and a major environmental issue. Therefore, it would be pertinent to study the effect of such organophosphate pesticides on long-term exposure by chronic studies to ascertain the residual toxicity. The fish silver barb (*Barbonymus gonionotus*) was selected as experimental model because of its wide availability in local tanks and ponds.

Hence, the present investigation was aimed at studying the histoarchitectural alterations in QP induced toxicity in some of the vital organs and blood parameters of the teleosts fish silver barb, so as to assess the damage and get an insight in its functional consequences.

## 2. Materials and Methods

Healthy and expected size of the silver barb (*B. gonionotus*) were obtained from the local fish farms and reared in the cemented tank. Individuals with a body weight of average weight 5.9 ± 3.61 g and standard length 8.11 ± 1.44 cm were selected and allowed to acclimatize to the laboratory conditions for two weeks to remove the suspected unhealthy subjects at 24.0–25.0°C. The experimental procedures were performed under the guidelines of the Animal Care Committee of Bangladesh Agricultural University. A total of 380 individuals were randomly assigned. They were housed in large water tank containing tap water provided with aeration system. Fish were fed twice a day with commercial dry pellets (Krishibid Fish Feed Ltd.) containing 38% protein. Feeding was discontinued 24 h prior to the experiment run and during the period of experiment. The water in the tank was changed once every two days. Fish were accepted as well as adapted to laboratory conditions when mortality less than 1% was recorded during acclimatization period of 14-day period. Fish of both sexes were used without discrimination.

The organophosphorus pesticide Quinalphos 25EC (O,O-diethyl O-quinoxaline-2-yl phosphorothioate, QP) was collected from the authorized dealer of the pesticide in original sealed container from Mymensingh, Bangladesh. The expiry date of the test substance checked prior to initiation of the treatment was found to be suitable for the exposure. The stock solution was prepared according to EC% active ingredient and desired concentrations of pesticides were poured carefully into 25 L of dechlorinated tap water in the test aquaria (45 × 30 × 30 cm^3^) by a micropipette and gently stirred with a glass rod to ensure complete mixing.

Before starting the test, all experimental aquaria were cleaned and filled with dechlorinated tap water. The acute toxicity test on silver barb with selected pesticide followed the OECD Directive number 203 “Fish, acute toxicity test.” The experimental water was kept in the tank for 24 h before QP was added. After performing range finding bioassay (0.002 to 20 ppm), a static acute toxicity bioassay was conducted according to the standard method to determine lethal concentration values (LC_10–100_) of QP. The experimental design incorporated eight groups (seven test groups and a control group) and replicates were prepared in the basic test. Each group was exposed to different concentrations like 1.5, 2.5, 4.0, 5.5, 7.0, 8.5, and 10 ppm for 96 h. Control fish were maintained in pesticide-free dechlorinated tap water in separate tank during this experiment. Exceeding aeration was applied to the aquarium for 2 h in order to obtain a homogeneous concentration of the toxic compound and randomly then ten fish were transmitted without stress to each test aquarium after proper acclimatization. Twenty-four hours before the experiment and during the toxicity test, feeding of the fish was stopped. Dead fish were removed immediately and mortality was recorded. Fish were considered dead when visible movement (e.g., operculum movements) ceased and there was no response to gentle probing of the caudal peduncle. The LC_0_, LC_50_, and LC_100_ values for the respective time intervals were determined by probit analysis.

Based on the result of the 96 h LC_50_ of QP, 200 fish of silver barb were exposed for 28 days to the nominal concentrations of 10% and 20% value of the LC_50_ (LC_50_ = 4.70 ppm) of the QP. Each concentration was replicated two times. There was a control group for each experiment. With the exception of the control tanks, appropriate volumes of the toxicant were added into each tank. Fish were fed to 3% body weight with 38% crude protein level pelleted diet. To maintain a relatively constant concentration of the toxicant and the level of dissolved oxygen as well as for minimizing the level of ammonia during the experiment, the toxicant and test waters were renewed at two-day intervals. Five fish were sampled from control and the concentrations at 7-day intervals until the end of 28 days.

Hematological analyses were carried out by standard methods suggested by Blaxhall and Daisley [6]. To obtain blood samples, fish were caught gently in a small scoop net and then quickly taken out from the water and held firmly on bench with a cloth covering the head and blood samples from each fish were withdrawn from caudal vein without anaesthetization by micropipette. Whole blood withdrawal process took less than one minute per fish which was considered important to avoid stress effects in order to minimize an error in normal blood values. Collected blood was gently pushed into a sterilized Eppendorf tube containing anticoagulant (ethylenediamine tetra-acetic acid, EDTA) to give a final concentration of 5 mg EDTA per cm^3^ blood. Blood samples were mixed gently and discarded if any difficulty was encountered in taking them or if clots were seen in the vial during inspection at the laboratory.

Blood glucose was measured by using a digital blood glucose kit (GLUCOLAB). Blood samples were taken at 1, 2, 3, 4, 7, 14, 21, and 28 days from 3 fish randomly selected from each treatment, a drop of blood sample was placed on the strips connected to the GLUCOLAB autocoding blood glucose test meter and results were recorded. The values were expressed in mmol/L. Hemoglobin (Hb) estimation was done by using a digital EasyLife Hb meter. The values were expressed in g/dL.

The microhematocrit method of Snieszko [7] was used to determine the hematocrit (PVC). Red blood cell (RBC) and white blood cell (WBC) counts were measured under light microscope with an improved Neubauer hemocytometer [8]. The derived hematological indices of mean corpuscular volume (MCV), mean corpuscular hemoglobin (MCH), and mean corpuscular hemoglobin concentration (MCHC) were calculated using standard formulae as described by Jain [9]:

 MCV = (PCV ÷ RBC  in  millions) × 10 *μ*m^3^,
 MCH = (Hb  in  g ÷ RBC  in  millions) × 10 pg,
 MCHC = (Hb ÷ PCV) × 100 g  per  100 mL.

All replicates were used for calculation of mean values. Statistics were performed with the SPSS 16.0 computer program (SPSS Inc., Chicago, Illinois, USA). Differences in mortality values and hematological parameters between different concentrations and between exposure times were processed statistically by means of the analysis of variance (one-way ANOVA). Lethal concentration values of QP were calculated using the probit analysis method. The lethal concentrations with 95% confidence limits were calculated.

## 3. Results

The range finding test carried out for concentration of QP between 0.002 ppm and 20 ppm for a period of 96 h showed no mortality up to concentration of 2 ppm while at 20 ppm 100% mortality was observed. Therefore, it was concluded that the median lethal concentration (LC_50_) of silver barb is between 2 and 20 ppm of QP. After performing range finding test, the median lethal toxicity study was determined for the concentration of QP ranging from 1.5 to 10.0 ppm. The exposure of fish to 96 h, at a concentration of 2.5 ppm showed 10% mortality, while at concentration of 10.0 ppm 100% mortality was noticed. The probit analysis showed that the lethal concentration for 50% mortality of the fish at 96 h was 4.70 ppm.

**Figure 1:**
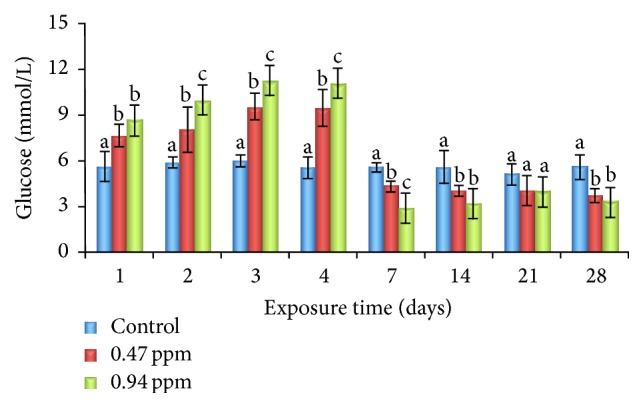
Effects of sublethal exposure of QP on glucose levels (Means ± SD) at different time intervals in silver barb. Different superscript alphabets are significantly different at *p* < 0.05.

In the present study, blood biochemical and hematological parameters were measured up to 28 days after starting the exposure of different sublethal dosages of QP. Our findings showed that the blood glucose levels were significantly increased at 1, 2, 3, and 4 days and decreased at 7, 14, 21, and 28 days of exposure periods compared to the control group (Figure 1).

The mean PCV, Hb, RBC, WBC, and derived erythrocyte indices (MCV, MCH, and MCHC) of silver barb exposed to chronic toxicity of QP are presented in Table 1. The alterations observed in hematological parameters were significant (*p* < 0.05) compared to the control. Significant variations (*p* < 0.05) were also observed between the various hematological parameters with different concentrations of toxicant.

**Figure 2:**
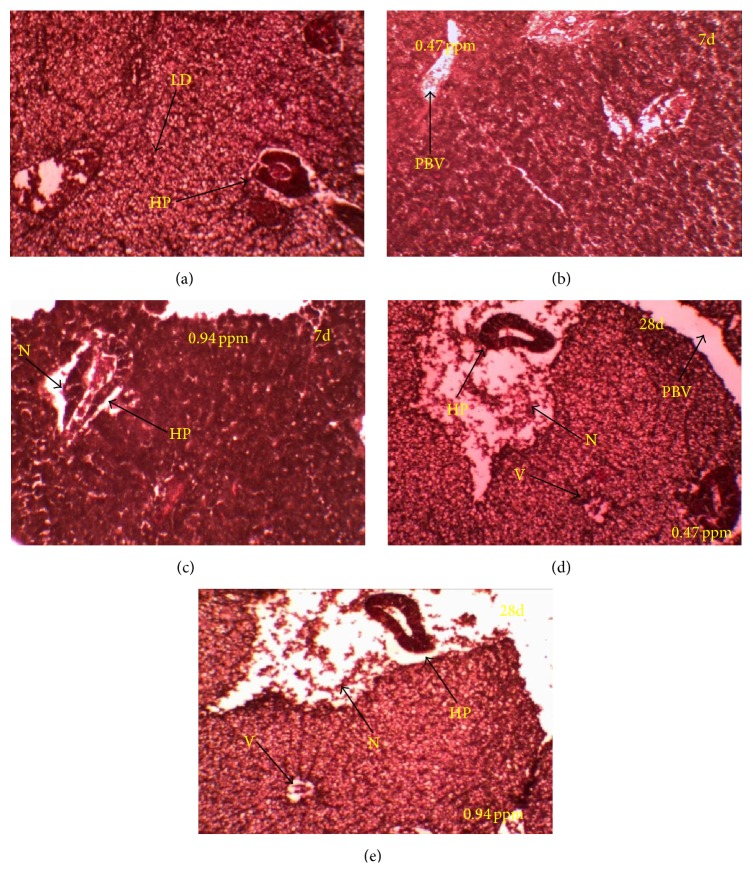
Histoarchitectural changes in liver (H & E stained, ×100) exposed to QP (a) control, (b) and (d) 0.47 ppm at 7 and 28 days, and (c) and (e) 0.94 ppm at 7 and 28 days. Arrows are indicating hepatopancreas (HP), lipid droplet (LD), necrosis (N), portal blood vessel (PBV), and vacuolation (V).

Histological studies revealed that the liver sections from control fish showed normal histoarchitecture; liver is characterized by polygonal shaped hepatocytes with granular cytoplasm. Hepatocytes were arranged in well-organized hepatic cords and separated by narrow blood sinusoids (Figure 2(a)). Liver of fishes exposed to 0.47 and 0.94 ppm QP for 7 days resulted in degeneration of cytoplasm in hepatocytes, rupture in blood vessels, and hypertrophy of hepatocytes and intravascular hemorrhage (Figures 2(b) and 2(c)) at 28 days resulted in cytoplasmic vacuolation in the hepatocytes and focal necrosis of hepatic tissue. Dead red blood cells were also seen in necrotic area (Figures 2(d) and 2(e)).

**Figure 3:**
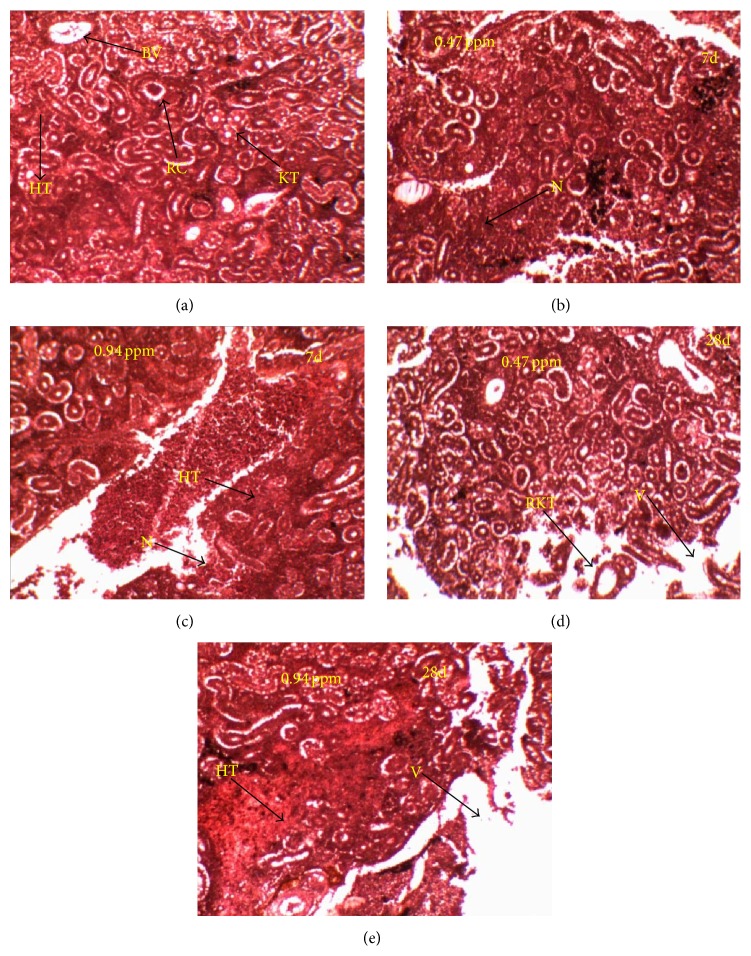
Histoarchitectural changes in kidney (H & E stained, ×100) exposed to QP (a) control, (b) and (d) 0.47 ppm at 7 and 28 days, and (c) and (e) 0.94 ppm at 7 and 28 days. Arrows are indicating blood vessel (BV), hematopoietic tissue (HT), kidney tubules (KT), necrosis (N), renal corpuscle (RC), ruptured kidney tubules (RKT), and vacuolation (V).

Kidney of control fish is composed of numerous renal corpuscles with well-developed glomeruli and a system of renal tubules (Figure 3(a)). Chronic sublethal QP exposed kidney sections showed several alterations such as degeneration of renal corpuscles, vacuolization, highly degenerated and distended of kidney tubules and hematopoietic tissue, changes in the nucleus structure, mild to severe necrosis, and hemorrhage (Figures 3(b)–3(e)).

## 4. Discussion

Blood offers important profile to study the toxicological impact on animal tissues. Different blood parameters are often subjected to change depending upon stress condition and various other environmental factors. Decrease or increase in certain blood parameters can be associated with the nature of species and the toxicants in different studies. The decrease in hematological variables (PCV, Hb, and RBC) of the exposed fish may be due to haemolysis and shrinkage of RBC by QP leading to significant decrease in hematocrit value which results in fish anemia. The increase rate of the breakdown of RBC or reduction rate of formation of RBCs might also be responsible for reduction in RBC count. Similar observations were reported for* Clarias gariepinus* treated with endosulfan pesticides [10]. This may also be attributed to hemodilution resulting from impaired osmoregulation across the gill epithelium [11]. Reduction in hematological indices may also be due to an appreciable decline in the hematopoiesis. Similar reduction in RBC was reported for cypermethrin treated* Labeo rohita* [12], African cat fish (*C. gariepinus*) treated with diazinon [13], and freshwater common carp (*Cyprinus carpio*) treated with atrazine [14]. In the present study, PCV value was significantly decreased with increasing the toxicity of QP at 28 days of exposure periods. Due to major reduction in RBC, PCV decreased. Similar to the present findings, Chindah et al. [15] found a decreased value of Hct in* Tilapia guineensis* to varying sublethal levels of cypermethrin and chlorpyrifos. Similar observations were also reported by Köprücü et al. [16], Jayaprakash and Shettu [17], and Jeyapriya et al. [18].

Increase in WBCs count occurred as a pathological response since these WBCs play a great role during infestation by stimulating the hemopoietic tissues and the immune system by producing antibodies and chemical substances working as defense against infection [19]. WBCs are important cells in the immune system, because of their main defensive function. The WBCs respond immediately to the change in medium due to toxicant. During toxic exposure period of QP, the WBC counts were enhanced. It indicates that fish can develop a defensive mechanism to overcome the toxic stress.

Reduction in Hb content of treated silver barb may be an indication of decline in Hb synthesis as well as reduction in oxygen carrying capacity which may perhaps be as a result of interference of QP with haem or globin synthesis pathway. Significant decrease (*p* < 0.05) in values of erythrocyte count, hematocrit, and Hb content compared to the control groups had been reported for catfish on acute exposure to diazinon [13]. The reduction in values obtained for hematological parameters of treated fish in this study showed that the physiological activities of the treated fish were affected.

Measurement of blood biochemical parameters is used as important diagnostic tool for the detection of abnormalities in the liver and other tissues [20]. Blood glucose has been shown to be a sensitive indicator of environmental stress for any chemical pollutant including pesticides [21]. The increase in value of glucose compared to the control indicated that silver barb generated more glucose to produce the energy used in combating the stress induced on the fish by QP. Increase in glucose level that was observed might have resulted from increase in glucogenesis and glycogenolysis as well as inhibition of glycogenolysis and glycogenesis during stress [22]. As the respiratory metabolism is being depressed, stored intracellular glycogen is utilized under such condition; the hyperglycemic hormone is released for the degradation of glycogen and glucose thus leaked into the blood causing hyperglycemia [23]. In the present investigation, sublethal exposure to QP at 0.47 and 0.94 ppm resulted in a significant increase in plasma glucose concentration, demonstrating the response of exposed fish to metabolic stress. Similar observations were also reported by Crestani et al. [24] in silver catfish (*Rhamdia quelen*), Velisek et al. [25] in common carp, and Adedeji [13] in cat fish (*C. gariepinus*).

Tissue histology is considered as an indicator of exposure to pollutants and represents a useful tool to assess the degree of pollution, particularly for sublethal and chronic effects [26]. The common liver abnormalities observed in the present study were hypertrophy of hepatocytes, mild to severe necrosis, blood spilling, ruptured central vein, lipid droplet, and vacuolation. And the changes in the liver were time and concentration dependent. Histological changes in the liver could be attributed to the fact that the liver is the major site of detoxification [27]; it is expected that the toxicant insecticide would reach there abundantly for detoxification and disposal [28]. The inability of fish to regenerate new liver cells may also have led to necrosis of hepatic cells of sinusoids. The present results were more or less in agreement with other studies in which necrosis and lipidosis vacuolization and an increase of macrophage aggregates and eosinophilic granular cells were recorded in fish treated with insecticides paraquat and malathion, respectively [29, 30].

The kidney of fish receives the largest proportion of postbranchial blood, and therefore renal lesions might be expected to be good indicators of environmental pollution. It has been found that the sublethal doses of QP induced alterations in the structure of kidney of silver barb. These results reveal that the histopathological changes are dose and duration dependent. The proximal tubules were the first to be affected resulting in the reduction of nuclear material in some cells. Then, the damage gradually spread through the glomeruli, hematopoietic tissues, and cells of distal and collecting tubules in that order. Similar changes have been reported in fish exposed to pesticides [31–33]. It is concluded that more or less similar pathological changes are induced in the kidney of different fish by different toxicants but the extent of damage varies depending upon the dose of toxicants, duration of exposure, toxicity of chemical, and susceptibility of the fish.

## 5. Conclusion

The hematological and histological changes that were taking place in the present study, as the initial period of exposure in the organs of the fish on exposure to QP toxicity, might be a part of defense mechanism. The further accumulation of QP in the organs of the fish on prolonged exposure caused significant hematological alterations and destruction in the organ structures. These changes may be potentially disruptive for the survivability of the silver barb in their natural environment. This fact should be taken into consideration when it is used for pest control in the agricultural fields surrounding their natural freshwater reservoirs.

## Figures and Tables

**Table 1:** Mean hematological parameters of silver barb exposed to sublethal concentrations (0.47 and 0.94 ppm) of QP in 1, 7, 14, 21, and 28 days.

Parameter	Exposure time (d)	Control (0.00 ppm)	Concentration of QP (ppm)	
			0.47	0.94
RBCs (×10^6^/mm^3^)	1	5.19 ± 0.54^a^	5.08 ± 0.33^a^	5.02 ± 21^a^
	7	5.21 ± 0.29^a^	4.67 ± 0.17^b^	3.88 ± 0.06^c^
	14	5.20 ± 0.44^a^	3.85 ± 0.11^b^	3.10 ± 0.11^c^
	21	5.27 ± 0.38^a^	2.40 ± 0.28^b^	2.20 ± 0.08^b^
	28	5.17 ± 0.28^a^	2.73 ± 0.06^b^	2.14 ± 0.08^c^

WBCs (×10^4^/mm^3^)	1	2.84 ± 0.13^a^	2.91 ± 0.17^a^	2.96 ± 0.19^a^
	7	2.90 ± 0.16^a^	3.62 ± 0.22^b^	4.37 ± 0.29^c^
	14	2.90 ± 0.24^a^	3.80 ± 0.68^b^	4.44 ± 0.21^c^
	21	2.92 ± 0.27^a^	4.31 ± 0.23^b^	5.16 ± 0.27^c^
	28	2.94 ± 0.07^a^	4.42 ± 0.28^b^	5.67 ± 0.12^c^

Hemoglobin (g/dL)	1	12.57 ± 0.23^a^	12.23 ± 0.26^a^	12.09 ± 0.57^a^
	7	12.63 ± 0.25^a^	10.73 ± 0.25^b^	10.27 ± 0.64^b^
	14	12.50 ± 0.30^a^	10.40 ± 0.75^b^	9.47 ± 0.60^c^
	21	12.67 ± 0.45^a^	9.13 ± 0.84^b^	8.90 ± 1.37^b^
	28	12.40 ± 0.56^a^	9.50 ± 0.61^b^	9.23 ± 0.55^b^

PCV (%)	1	46.22 ± 2.16^a^	45.31 ± 1.52^a^	44.49 ± 2.67^a^
	7	46.90 ± 3.44^a^	32.14 ± 0.56^b^	27.20 ± 6.05^b^
	14	46.23 ± 1.29^a^	27.16 ± 2.63^b^	26.27 ± 2.77^b^
	21	43.16 ± 4.44^a^	24.91 ± 3.13^b^	24.88 ± 4.59^b^
	28	44.31 ± 3.98^a^	22.75 ± 3.13^b^	22.19 ± 2.45^b^

MCV (*μ*m^3^)	1	89.05 ± 4.12^a^	89.19 ± 3.21^a^	88.62 ± 4.54^a^
	7	90.33 ± 10.58^a^	68.88 ± 3.09^b^	69.90 ± 14.48^b^
	14	89.41 ± 10.00^a^	70.49 ± 5.41^b^	84.99 ± 7.20^a^
	21	82.46 ± 12.87^a^	103.93 ± 10.16^b^	112.62 ± 18.27^b^
	28	85.55 ± 3.12^a^	83.42 ± 12.49^a^	103.79 ± 11.79^c^

MCH (pg)	1	24.21 ± 1.89^a^	24.07 ± 1.66^a^	24.08 ± 1.97^a^
	7	24.28 ± 1.29^a^	22.99 ± 0.47^b^	26.43 ± 1.29^c^
	14	24.17 ± 2.63^a^	27.05 ± 2.40^b^	30.54 ± 1.58^c^
	21	24.09 ± 1.36^a^	38.65 ± 8.34^b^	40.58 ± 7.65^b^
	28	24.01 ± 1.66^a^	34.84 ± 2.96^b^	43.15 ± 2.13^c^

MCHC (%)	1	27.19 ± 1.43^a^	26.99 ± 1.12^a^	27.17 ± 3.13^a^
	7	27.03 ± 1.86^a^	33.41 ± 1.30^b^	38.78 ± 7.37^b^
	14	27.12 ± 0.22^a^	38.68 ± 6.16^b^	36.19 ± 4.69^b^
	21	29.63 ± 4.10^a^	37.31 ± 8.10^a^	37.16 ± 12.3^a^
	28	28.14 ± 2.92^a^	42.2 ± 5.61^b^	41.78 ± 2.84^b^

Values are mean ± standard deviation, *n* = 5, different alphabetic superscripts (a, b, and c) indicate significant differences at *p* < 0.05 level, RBC = red blood cell, WBC = white blood cell, Hb = hemoglobin, PCV = packed cell volume, MCV = mean corpuscular volume, MCH = mean corpuscular hemoglobin, and MCHC = mean corpuscular hemoglobin concentration.
